# Difficult airway management of a patient with limited range of motion in the temporomandibular joint and cervical extension caused by psoriatic arthritis: a case report

**DOI:** 10.1186/s40981-020-00351-6

**Published:** 2020-06-08

**Authors:** Makishi Maeda, Tomohiro Chaki, Ryoichi Kawaguchi, Tomohiko Kimijima, Michiaki Yamakage

**Affiliations:** Department of Anesthesiology, Sapporo Medical University School of Medicine, 291, South 1, West 16, Chuo-ku, Sapporo, Hokkaido Japan

**Keywords:** Airway management, Psoriasis, Psoriatic arthritis, Temporomandibular joint

## Abstract

**Background:**

Psoriasis vulgaris, a chronic inflammatory skin disease, rarely causes temporomandibular arthritis. We report a case of difficult airway management of a patient with limited range of motion in the temporomandibular joint and cervical extension caused by psoriatic arthritis.

**Case presentation:**

A 33-year-old man was scheduled to undergo laparoscopic colectomy. On admission, he was diagnosed with psoriatic arthritis. After induction of general anesthesia, we attempted intubation using Pentax Airway Scope® with a thin intlock blade and using a bronchoscope, but it was impossible because of the limited oral space and mandibular elevation. Because of concerns about cannot intubate, cannot ventilate, we antagonized the neuromuscular block and he emerged from general anesthesia. Finally, we succeeded in awake intubation via the nasal cavity using a bronchoscope under spontaneous respiration.

**Conclusions:**

Although psoriasis vulgaris is very rarely associated with temporomandibular arthritis, anesthesiologists should consider that it can cause perioperative difficult airways.

## Background

Psoriasis vulgaris, a chronic inflammatory skin disease, is occasionally associated with various conditions such as arthritis, cardiovascular disease, metabolic syndrome, inflammatory bowel disease, and depression. Although the etiology has not been fully clarified, it is thought that genetic predisposition, environmental factors, and autoimmune mechanisms are involved [1]. Psoriatic arthritis (PsA) is an arthritis similar to rheumatoid arthritis. However, rheumatoid factors are negative in many cases and affected patients are younger and more likely to be male [2]. A small number of joints are asymmetrically affected, and the distal interphalangeal joint, knee joint, and spine are common sites. PsA in rare cases causes temporomandibular arthritis, for which airway management during general anesthesia may be difficult [3]. We report a case of difficult airway management of a patient with limited range of motion in the temporomandibular joint (TMJ) and cervical extension caused by PsA.

## Case presentation

**Fig. 1:**
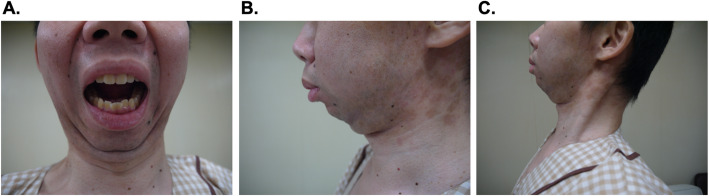
Preoperative airway evaluation. The patient showed **a** limited mouth opening, presence of teeth, **b** micrognathia, **c** limited thyroid-mental distance, and neck extension. Maximum neck extension is shown in picture **c**

A 33-year-old man was diagnosed with colon cancer after melena and was scheduled to undergo laparoscopic colectomy. His height was 166 cm and his weight was 57 kg. At the age of 17 years, a scaly erythema rash appeared on the front of his lower leg and gradually spread throughout his body. He visited a dermatologist at the age of 19 years and was diagnosed with psoriasis vulgaris. He received topical therapy (He could not remember what drugs were used.) and systemic therapy with betamethasone at 1 mg/day for treatment of psoriasis vulgaris. However, the treatment was discontinued at his discretion several months ago. He has been aware of right knee pain for 3 years, but he had not been diagnosed or treated for joint symptoms. On admission, he had exacerbation of knee pain and was examined by an orthopedist. Preoperative laboratory data showed elevations of C-reactive protein (10.05 mg/dL) and matrix metalloproteinase-3 (1996.5 ng/mL), but there were no other abnormal data including rheumatoid factor. Because he had pain and swelling in his right knee and his rheumatoid factor was negative, he was diagnosed with PsA by the CASPAR (ClASsification criteria for Psoriatic ARthritis) criteria [2]. To meet the CASPAR criteria (Table 1), a patient must have inflammatory articular disease with ≥ 3 points from the 5 categories. He had 3 points by current psoriasis (2 points) and negative rheumatoid factor (1 point). He was also aware that it had gradually become more difficult to open his mouth in the past a few years. Drinking water was possible with no problem, but he had to eat his food in small portions. However, he did not visit a hospital and his TMJ had not been evaluated by a dentist. Preoperative airway evaluation by an anesthesiologist included multiple difficult airway predictors: limited mouth opening (1.5 transverse fingers), presence of teeth, limited thyromental distance (thyroid incision distance of 4.5 cm), limited neck extension, Mallampati III, limited mandibular elevation (upper lip bite test III degree), and micrognathia (Fig. 1).

**Table 1:** The CASPAR (ClASsification criteria for Psoriatic ARthritis) criteria

Category	Points
1. Medical history of psoriasis*	
Current psoriasis	2
Personal history of psoriasis or family history of psoriasis	1
2. Nail dystrophy	
Typical psoriatic nail dystrophy observed in current physical examination	1
3. Rheumatoid factor	
A negative test result for the presence of rheumatoid factor	1
4. Dactylitis	
Current dactylitis or a history of dactylitis	1
5. Radiographic evidence	
Juxta articular new bone formation on plain radiographs of the hand or foot	1

To meet the CASPAR criteria, a patient must have inflammatory articular disease (joint, spine, or entheseal) with ≥ 3 points from the 5 categories

*In this category, only either can be selected

No premedication was administered. Because of extensive psoriasis lesions on his back, epidural anesthesia was not performed to avoid infection at the puncture site. We considered that mask ventilation might be possible. After oxygenation with oxygen at 6 L/min, rapid induction of general anesthesia was performed using 100 mg propofol and 50 mg rocuronium. Although it was somewhat difficult, mask ventilation was possible by using an oral airway. The mouth opening disorder did not improve after administration of rocuronium. First, we attempted intubation using Pentax Airway Scope® (AWS) (Pentax, Tokyo, Japan) with a thin intlock blade. Although the tip of the intlock blade could be inserted into the oral cavity, it was impossible to insert it up to the glottis because of the limited oral space and the presence of teeth. Next, we attempted oral intubation using a bronchoscope (Olympus, Tokyo, Japan). However, it was impossible even by multiple anesthesiologists because mandibular elevation was difficult and the visual field was poor. As the procedure was repeated, mask ventilation gradually became more difficult, and we were concerned about the risk of becoming “cannot intubate cannot ventilate (CICV).” Therefore, we decided to antagonize the neuromuscular block using 600 mg sugammadex and we awoke him. After confirming his arousal and spontaneous respiration, we succeeded in awake intubation via the nasal cavity using a bronchoscope. We used a cuffed 6.5-mm tracheal tube (Parker Medical, Bridgewater, MA) and inserted it 27 cm from his nose. Because he was given 150 μg of fentanyl and he did not seem to suffer, he did not complain of recall during tracheal intubation. During the airway management, oxygen saturation did not drop below 90%. General anesthesia was maintained with fentanyl, remifentanil, and desflurane. After the operation had been completed, we confirmed that he could follow our instructions and that he had steady spontaneous respiration and adequate neuromuscular block reversal. Then extubation was carefully performed. After extubation, his breathing remained stable and he was able to breathe deeply and cough. There were no breathing or airway-related problems or complications in his postoperative course.

## Discussion

Psoriasis vulgaris is a chronic inflammatory skin disease with keratinous erythema as the main feature. The worldwide prevalence is 0.09–11.4% [4, 5]. The value has a very wide range because there are differences in prevalence depending on the region. It is less common in Asia and Africa and more common in Europe and the USA. For example, the prevalence rate in Japan was reported to be 0.34% [6]. Although the etiology has not been fully clarified, it is thought that genetic predisposition, environmental factors, and autoimmune mechanisms are involved. In addition to skin symptoms, various conditions such as arthritis, cardiovascular disease, metabolic syndrome, inflammatory bowel disease, and depression can be complicated [1]. For example, psoriasis is an independent risk factor for ischemic heart disease [7]. Recently, an association of psoriasis vulgaris with abdominal aortic aneurysm and aortic stenosis was reported, and its pathophysiology is thought to be an inflammatory mechanism similar to that for atherosclerotic lesions [8]. Therefore, we should consider that psoriasis vulgaris is not just a skin disease but a systemic inflammatory disease that can cause various complications. Conventional therapies for treatment of psoriasis include topical therapy, phototherapy, and systemic therapy. Systemic drugs include methotrexate, ciclosporin, and glucocorticosteroids. In the past decade, several biologics have been developed and approved.

PsA is an arthritis associated with psoriasis that occurs in about 1.3–34.7% of patients with psoriasis vulgaris [9, 10]. PsA affects a small number of joints asymmetrically, and the distal interphalangeal joint, knee joint, and spine are common sites. Common symptoms are stiffness, pain, swelling, and tenderness of the joints. It may have clinical symptoms similar to those of rheumatoid arthritis, but as in this case, more than 90% of the cases are negative for rheumatoid factor. The CASPAR criteria presented by Taylor in 2006 are often used for diagnosis [2].

PsA of the TMJ, which was a problem in this case, is exceedingly rare and only about 40 cases have been reported. Erosion, bony proliferation, bone surface flattening, and sclerosis are typical changes, and conventional radiography and a computed tomography scan are the most widely used methods for diagnosing PsA of the TMJ [3]. Our patient decided by himself to discontinue treatment, and arthritis was not diagnosed or treated until hospitalization. Therefore, temporomandibular arthritis may have progressed. In our case, arthritis was diagnosed by an orthopedist for knee pain, but the TMJ and cervical movement were not evaluated before the operation. There has been no report from an anesthesiology department that psoriasis caused a difficult airway, and we did not recognize it in the preoperative period.

Airway problems during induction of anesthesia can lead to death, and careful preoperative evaluation and anesthesia planning are therefore important. In this case, several difficult airway predictors were recognized preoperatively. According to previous studies, Mallampati III, jaw protrusion-limited, male sex, presence of teeth, limited neck extension, and limited thyromental distance were shown to be predictors of a difficult airway. The probability of difficult mask ventilation was estimated to be 2.7% [11], and that of difficult mask ventilation combined with difficult laryngoscopy was estimated to be 1.69% [12]. Therefore, we prepared video laryngoscopes such as AWS and a bronchoscope. However, even after administration of rocuronium, the mouth opening disorder did not improve, and it was impossible to insert the AWS intlock blade up to the glottis. Moreover, mobility of his TMJ did not change and it was difficult to raise the mandibular elevation. The risk of CICV increases when repeated intubations are attempted multiple times or with multiple devices [13]. In this case as well, gradually mask ventilation became more difficult, and there was concern about upper airway and glottal edema due to multiple airway management operations. Therefore, according to guidelines for airway management [14], CICV was avoided by antagonizing the neuromuscular block, restoring consciousness, restoring patency of the airway, and resuming spontaneous respiration. The reason for the finally successful nasal intubation was that it is easier to maintain the bronchoscope in midline compared to that in the case of oral intubation, and the upper airway muscle tone is maintained after awakening. Thus, awake intubation via the nasal cavity using a bronchoscope may be a useful procedure for airway maintenance in patients with temporomandibular arthritis.

We experienced a case of difficult airway management of the patient who had range of motion of the limited TMJ and cervical extension caused by psoriatic arthritis. Although psoriasis vulgaris is very rarely associated with temporomandibular arthritis, anesthesiologists should consider that it can cause perioperative difficult airways.

## Data Availability

The datasets are available from the corresponding author on reasonable request.

## Abbreviations

AWS: Airway Scope
CASPAR: ClASsification criteria for Psoriatic ARthritis
CICV: Cannot intubate, cannot ventilate
PsA: Psoriatic arthritis
TMJ: Temporomandibular joint
